# Long-term prognostic value of the GenesWell BCT score in Asian women with hormone receptor-positive/HER2-negative early breast cancer

**DOI:** 10.1007/s12282-023-01509-7

**Published:** 2023-10-09

**Authors:** Yoshitaka Fujiki, Masahiro Kashiwaba, Mutsumi Sato, Junko Kawano, Megumi Teraoka, Shuichi Kanemitsu, Yoshiaki Rai, Tetsuhiko Taira, Yoshiaki Sagara, Yasuyo Ohi, Uiree Jo, Young-Won Lee, Sae Byul Lee, Gyungyub Gong, Young Kee Shin, Mi Jeong Kwon, Yasuaki Sagara

**Affiliations:** 1 Department of Breast and Endocrine Surgical Oncology, Hakuaikai Social Medical Corporation, Sagara Hospital, 3-28 Matsubara, Kagoshima, 892-0098 Japan; 2 Department of Medical Oncology, Hakuaikai Social Medical Corporation, Sagara Hospital, Kagoshima, Japan; 3 Department of Radiology, Hakuaikai Social Medical Corporation, Sagara Hospital, Kagoshima, Japan; 4 Department of Pathology, Hakuaikai Social Medical Corporation, Sagara Hospital, Kagoshima, Japan; 5 Department of Pathology, University of Ulsan College of Medicine, Asan Medical Center, Seoul, Republic of Korea; 6 Division of Breast Surgery, Department of Surgery, University of Ulsan College of Medicine, Asan Medical Center, Seoul, Republic of Korea; 7 Laboratory of Molecular Pathology and Cancer Genomics, Research Institute of Pharmaceutical Sciences and College of Pharmacy, Seoul National University, Seoul, Republic of Korea; 8 Department of Molecular Medicine and Biopharmaceutical Sciences, Graduate School of Convergence Science and Technology, Seoul National University, Seoul, Republic of Korea; 9 Vessel-Organ Interaction Research Center, College of Pharmacy, Kyungpook National University, 80 Daehak-Ro, Buk-Gu, Daegu, 41566 Republic of Korea; 10 BK21 FOUR Community-Based Intelligent Novel Drug Discovery Education Unit, College of Pharmacy and Research Institute of Pharmaceutical Sciences, Kyungpook National University, Daegu, Republic of Korea

**Keywords:** GenesWell BCT score, Hormone receptor-positive/HER2-negative breast cancer, Prognostic value, Late recurrence, Asian women

## Abstract

**Background:**

Accurate prediction of the risk of recurrence is crucial for optimal treatment decisions in hormone receptor (HR)-positive/human epidermal growth factor receptor 2 (HER2)-negative early breast cancer. The GenesWell BCT is a molecular assay to predict the 10-year risk of distant metastasis. In this study, we evaluated the long-term prognostic value of the GenesWell BCT assay.

**Methods:**

The BCT score was assessed in patients with HR-positive/HER2-negative early breast cancer who did not receive chemotherapy. We compared the 15-year distant metastasis-free survival (DMFS) between risk groups classified based on the BCT score. The risk of early (0–5 years) and late (5–15 years) recurrence was evaluated based on the BCT score classification.

**Results:**

According to the BCT score, 366 patients from Japan and Korea were categorized as BCT low risk (83.6%) and high risk (16.4%) for distant metastasis. Median follow-up time was 17.4 years. The 15-year DMFS rate was significantly lower in the BCT high-risk group (63.3%) than in the BCT low-risk group (93.6%) (*P* < 0.001). The BCT risk group was an independent prognostic factor for 15-year DMFS (hazard ratio, 4.59; 95% confidence interval 2.13–9.88; *P* < 0.001). Furthermore, the BCT score was a significant predictor of late recurrence (5–15 years) in patients aged ≤ 50 years and those aged > 50 years, and added prognostic information to traditional clinical prognostic factors.

**Conclusion:**

The BCT score can identify patients at low risk for recurrence who may not require adjuvant chemotherapy or extended endocrine therapy, regardless of age.

**Supplementary Information:**

The online version contains supplementary material available at 10.1007/s12282-023-01509-7.

## Introduction

Breast cancer is the most common cancer type and the leading cause of cancer-related deaths in women worldwide [1]. In particular, the incidence of breast cancer has been rapidly increasing in Asian countries, including the Republic of Korea and Japan, which have had historically low incidence rates [2]. Changes in the lifestyle and sociocultural environments of Asian women, such as low birth rates, increased prevalence of overweight or obesity, and reduced physical activity, are considered responsible for the high prevalence of breast cancer [1].

Hormone receptor (HR)-positive/human epidermal growth factor receptor 2 (HER2)-negative breast cancer, which accounts for 50–60% of all breast cancers, has better prognosis than other breast cancer subtypes [3]. However, women with HR-positive/HER2-negative early breast cancer have a higher risk of late recurrence beyond 5 years after primary endocrine therapy than those with HR-positive/HER2-positive or HR-negative breast cancer [4–6]. Therefore, accurate prediction of the risk of late recurrence in this population is crucial for making optimal treatment decisions 5 years after endocrine therapy (i.e., to extend endocrine therapy or administer adjuvant chemotherapy). Several molecular assays based on multigene signatures, such as Oncotype DX, MammaPrint, Prosigna, and EndoPredict, have been developed to predict the risk of recurrence in early breast cancer [7]. Some multigene signatures (Prosigna Risk of Recurrence, Breast Cancer Index, and EndoPredict score) have been shown to be strong predictors of late recurrence 5–10 years after diagnosis [8–12]. However, late recurrence can occur beyond the 10-year time point. Women with estrogen receptor (ER)-positive early breast cancer who received 5 years of endocrine therapy had a persistent risk of recurrence of and death from breast cancer for at least 20 years after diagnosis, highlighting the long-term risk of recurrence in patients with this breast cancer subtype [13]. Therefore, additional studies with longer follow-up periods are needed to determine the long-term prognostic value of molecular assays and consequently improve adjuvant treatment decision-making in breast cancer.

Although the prognostic and predictive values of the Oncotype DX 21-gene recurrence score (RS) have been validated in White women, it is unclear whether the 21-gene RS predicts patient prognosis and benefit of chemotherapy in other populations, including Asian women with early breast cancer. Furthermore, some studies have reported differences in clinical outcomes between races within the risk groups classified by the 21-gene RS [14, 15]. Recently, a population-based study using the Surveillance, Epidemiology, and End Results (SEER) database demonstrated that the 21-gene RS significantly predicts chemotherapy benefits in White women, but not in Black and Asian American/Pacific Islander women, suggesting a racial difference in the predictive value of this score for chemotherapy benefit [16].

The GenesWell Breast Cancer Test (BCT) (Gencurix, Inc., Seoul, Republic of Korea) is a molecular assay that predicts the risk of distant metastasis in early breast cancer based on the BCT score calculated from the expression of six prognostic genes in combination with two clinical factors [17]. Previously, we demonstrated that the BCT score is a significant predictor of 10-year distant metastasis as well as of both early (0–5 years) and late (5–10 years) distant metastasis in Asian women with HR-positive/HER2-negative early breast cancer [17]. The BCT score also predicted the benefit of chemotherapy in Asian women with early breast cancer [18]. A recent study [19] further validated the prognostic value of the BCT score in predicting the risk of 10-year distant metastasis in patients aged ≤ 50 years as well as in those aged > 50 years, suggesting that it can be used to identify patients at low risk of recurrence who will not benefit from adjuvant chemotherapy, regardless of their age. However, to date, the prognostic value of the BCT score has only been evaluated for predicting the risk of 10-year distant metastasis.

In this study, we reassessed the prognostic value of the GenesWell BCT assay in Asian women with longer term follow-up. The 15-year distant metastasis-free survival (DMFS) rates for women with HR-positive/HER2-negative early breast cancer from Korean and Japanese sites were compared according to their risk stratification based on the BCT score. We further evaluated the risk of late recurrence 5–15 years after surgery based on the BCT score classification.

## Materials and methods

### Patients and tumor samples

This study included patients with HR-positive/HER2-negative early breast cancer from two cohorts (Korea and Japan) who received endocrine therapy without chemotherapy. For the Japanese cohort, consecutive 149 patients with pT1–2, pN0–1, and HR-positive/HER2-negative early breast cancer who had undergone surgery at the Sagara Hospital (Kagoshima, Japan) between 2003 and 2007 were screened. Formalin-fixed, paraffin-embedded (FFPE) tumor samples and clinicopathological information such as tumor size, nodal status, and histologic grade were collected. Of the 149 FFPE tumor samples, four with insufficient RNA quality and one from patient not meeting the sample criteria were excluded, leaving 144 samples for survival analysis. In the Korean cohort, 222 women with HR-positive/HER2-negative early breast cancer who underwent curative resection of the primary tumor at Asan Medical Center (AMC) (Seoul, Republic of Korea) and had a reportable BCT score analyzed in our previous study [17] were included. Their 15-year follow-up clinical information was obtained.

This study was approved by the Institutional Review Boards of the Sagara Hospital (18–40) and AMC (2022–1700) and was performed in accordance with the Declaration of Helsinki. Because the study was retrospective, and patient information was anonymized and de-identified prior to analysis, the requirement for informed consent was waived. General consent to use patient samples for research purposes was obtained from patients at the time of surgery.

### GenesWell BCT assay

Total RNA was isolated from FFPE tumor samples, and the GenesWell BCT assay based on quantitative real-time reverse transcription-polymerase chain reaction was performed as previously described [17]. The BCT score was calculated from the relative expression values of six prognostic genes (*UBE2C*, *TOP2A*, *RRM2*, *FOXM1*, *MKI67,* and *BTN3A2*) normalized by three reference genes (*CTBP1*, *CUL1*, and *UBQLN1*) and two clinical variables (tumor size and pathologic nodal status). Patients were categorized into the BCT high-risk group if their BCT score was ≥ 4 and into the BCT low-risk group if their BCT score was < 4 as previously described [17].

### Statistical analyses

The primary endpoint was 15-year DMFS. DMFS was defined as the time from the date of surgery to the date of the finding of distant metastasis. The Kaplan–Meier method was used to estimate the probability of DMFS according to the BCT risk category, and the statistical differences between risk groups were compared using the log-rank test. Univariate and multivariate Cox analyses were used to assess the prognostic value of the BCT score on the risk of distant metastasis. A likelihood ratio test was used to evaluate the added prognostic value of the BCT score to traditional clinical prognostic factors.

All hazard ratios were reported with 95% confidence intervals (CIs). All statistical tests were two-sided, and a *P* value < 0.05 was regarded as statistically significant. Statistical analyses were performed using R 4.1.2 (http://www.R-project.org).

## Results

### Patient characteristics

A total of 366 patients, including 144 from the Sagara Hospital and 222 from the AMC, were included in this retrospective analysis. The patient characteristics are summarized in Table 1. In the total cohort, the median age was 52 years (range 29–80 years), and 45.9% of patients were aged ≤ 50 years. Notably, in the Sagara Hospital, the percentage of patients aged > 50 years (63.9%) was higher than that of patients aged ≤ 50 years (36.1%), while the percentages of patients in these two age groups in the AMC cohort were similar. Overall, 83.6% (306/366) of patients were categorized as BCT low risk and 16.4% (60/366) as BCT high risk according to the BCT score. Most patients had lymph node-negative tumors (91.0%) and small tumor size (≤ 2 cm) (82.0%). Among women with node-negative breast cancer (*n* = 333), 88.0% (293/333) were categorized into the BCT low-risk group. In contrast, only 39.4% (13/33) of women with node-positive breast cancer were categorized into the BCT low-risk group. A similar distribution of the BCT risk groups was observed in both the Japanese and Korean cohorts. Moreover, in both cohorts, the BCT high-risk group was significantly correlated with unfavorable clinical factors such as positive nodal status (*P* < 0.001) and larger tumor size (> 2 cm) (*P* < 0.001) (Table 1). There were no significant differences in age and histologic grade between the two risk groups in the Japanese cohort, whereas the BCT high-risk group was significantly associated with older age (> 50 years) (*P* < 0.001) and higher histologic grade (*P* < 0.001) in the Korean cohort.

**Table 1:** Patient characteristics according to the BCT risk group

	Sagara Hospital + AMC				Sagara Hospital (Japan)				AMC (Korea)			
	Total	BCT low risk	BCT high risk	*P* value	Total	BCT low risk	BCT high risk	*P* value	Total	BCT low risk	BCT high risk	*P* value
*n* (%)	366 (100.0%)	306 (100.0%)	60 (100.0%)		144 (100.0%)	119 (100.0%)	25 (100.0%)		222 (100.0%)	187 (100.0%)	35 (100.0%)	
Age				** < 0.001**^**a**^				1.000^a^				** < 0.001**^**a**^
≤ 50 years	168 (45.9%)	152 (49.7%)	16 (26.7%)		52 (36.1%)	43 (36.1%)	9 (36.0%)		116 (52.3%)	109 (58.3%)	7 (20.0%)	
> 50 years	198 (54.1%)	154 (50.3%)	44 (73.3%)		92 (63.9%)	76 (63.9%)	16 (64.0%)		106 (47.7%)	78 (41.7%)	28 (80.0%)	
Positive nodes				** < 0.001**^**b**^				** < 0.001**^**b**^				** < 0.001**^**b**^
0	333 (91.0%)	293 (95.8%)	40 (66.7%)		130 (90.3%)	113 (95.0%)	17 (68.0%)		203 (91.4%)	180 (96.3%)	23 (65.7%)	
1	23 (6.3%)	9 (2.9%)	14 (23.3%)		10 (6.9%)	5 (4.2%)	5 (20.0%)		13 (5.9%)	4 (2.1%)	9 (25.7%)	
2	5 (1.4%)	1 (0.3%)	4 (6.7%)		2 (1.4%)	0 (0.0%)	2 (8.0%)		3 (1.4%)	1 (0.5%)	2 (5.7%)	
3	5 (1.4%)	3 (1.0%)	2 (3.3%)		2 (1.4%)	1 (0.8%)	1 (4.0%)		3 (1.4%)	2 (1.1%)	1 (2.9%)	
Tumor size				** < 0.001**^**a**^				** < 0.001**^**b**^				** < 0.001**^**a**^
≤ 2 cm	300 (82.0%)	275 (89.9%)	25 (41.7%)		116 (80.6%)	105 (88.2%)	11 (44.0%)		184 (82.9%)	170 (90.9%)	14 (40.0%)	
> 2 cm	66 (18.0%)	31 (10.1%)	35 (58.3%)		28 (19.4%)	14 (11.8%)	14 (56.0%)		38 (17.1%)	17 (9.1%)	21 (60.0%)	
Histologic grade				** < 0.001**^**a**^				0.125^b^				** < 0.001**^**a**^
1	97 (26.5%)	89 (29.1%)	8 (13.3%)		61 (42.4%)	53 (44.5%)	8 (32.0%)		36 (16.2%)	36 (19.3%)	0 (0.0%)	
2	227 (62.0%)	193 (63.1%)	34 (56.7%)		79 (54.9%)	64 (53.8%)	15 (60.0%)		148 (66.7%)	129 (69.0%)	19 (54.3%)	
3	42 (11.5%)	24 (7.8%)	18 (30.0%)		4 (2.8%)	2 (1.7%)	2 (8.0%)		38 (17.1%)	22 (11.8%)	16 (45.7%)	

AMC, Asan Medical Center

^a^Chi-square test; ^b^Fisher’s exact test

*P* values < 0.05 are marked in bold

### Long-term prognostic value of the BCT score

**Fig. 1:**
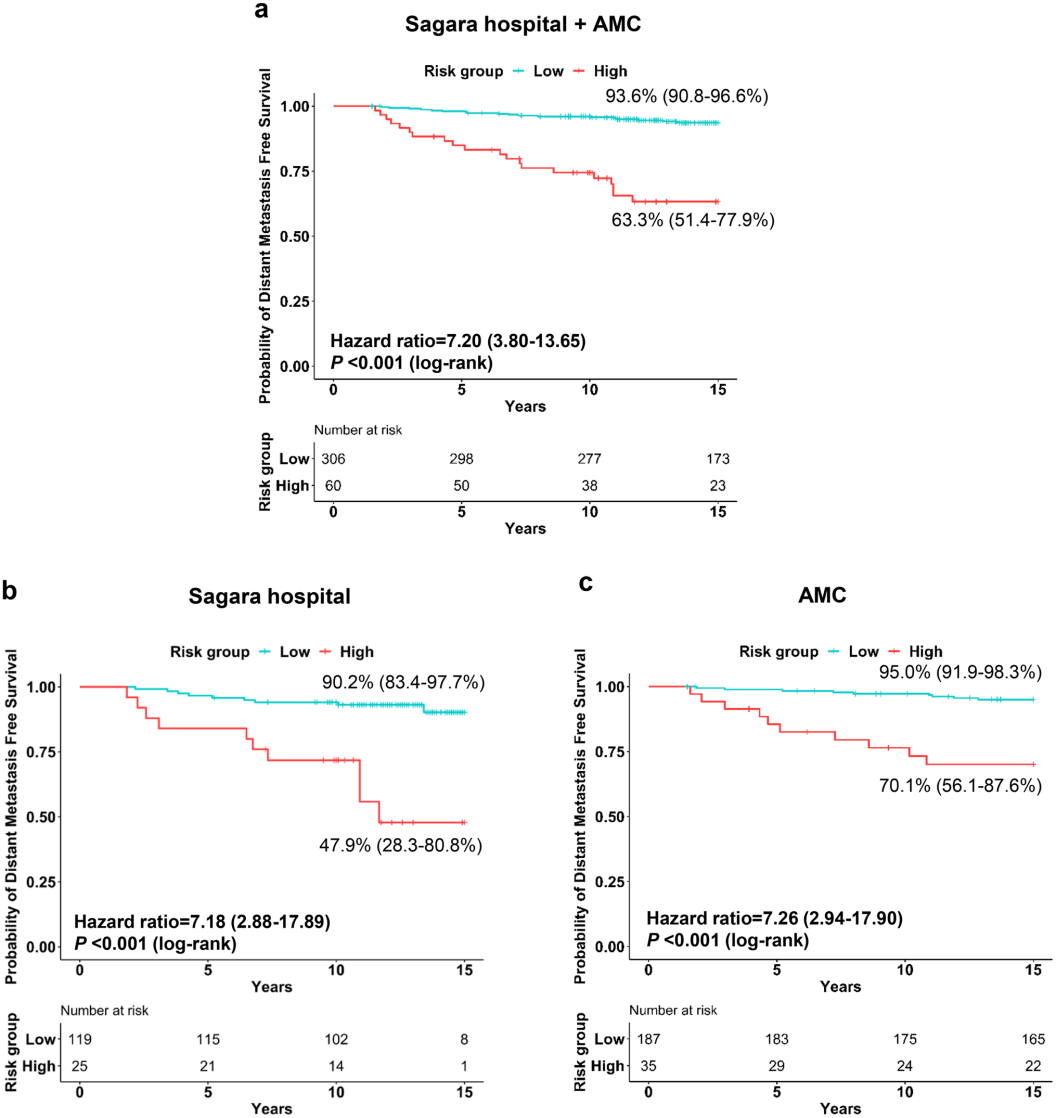
Kaplan–Meier plots of 15-year distant metastasis-free survival according to the BCT risk group. **a** Total, **b** Sagara Hospital (Japan), and **c** Asan Medical Center (AMC) (Korea) cohorts

In the total cohort, the maximum and median follow-up time was 24.1 and 17.4 years, respectively. Kaplan–Meier survival analysis showed a significantly shorter DMFS of patients in the BCT high-risk group than in the BCT low-risk group (*P* < 0.001) (Fig. 1a). The probabilities of 15-year DMFS for patients in the BCT low-risk and high-risk groups were 93.6% (95% CI, 90.8–96.6%) and 63.3% (51.4–77.9%), respectively. We then evaluated the association between the BCT score and patient survival using Cox proportional hazard model. In the univariate analysis for DMFS in the total cohort, the BCT high-risk group was significantly associated with an increased risk of 15-year distant metastasis (hazard ratio, 7.20; 95% CI, 3.80–13.65; *P* < 0.001) (Table 2). Tumor size, nodal status, histologic grade, and age were also significant predictors of distant metastasis. In the multivariate analysis, the BCT risk group remained a statistical significance after adjusting for clinical factors (hazard ratio, 4.59; 95% CI, 2.13–9.88; *P* < 0.001) (Table 2), demonstrating that the BCT high-risk group is a negative independent predictor of 15-year distant metastasis.

**Table 2:** Univariate and multivariate analyses for 15-year distant metastasis-free survival

	Sagara Hospital + AMC					
	Univariate			Multivariate		
	Hazard ratio	95% CI	*P* value	Hazard ratio	95% CI	*P* value
BCT risk (low vs. high)	7.20	3.80–13.65	** < 0.001**	4.59	2.13–9.88	** < 0.001**
Tumor size (≤ 2 cm vs. > 2 cm)	4.33	2.28–8.22	** < 0.001**	1.72	0.81–3.67	0.161
Positive nodes (0–3)	1.79	1.19–2.70	**0.005**	1.05	0.63–1.77	0.839
Histologic grade (1 vs. 2/3)	3.12	1.11–8.80	**0.031**	2.18	0.76–6.26	0.146
Age (≤ 50 vs. > 50 years)	2.05	1.03–4.06	**0.041**	1.38	0.67–2.86	0.383

	Sagara Hospital (Japan)					
	Univariate			Multivariate		
	Hazard ratio	95% CI	*P* value	Hazard ratio	95% CI	*P* value
BCT risk (low vs. high)	7.18	2.88–17.89	** < 0.001**	3.92	1.38–11.14	**0.010**
Tumor size (≤ 2 cm vs. > 2 cm)	3.73	1.50–9.31	**0.005**	1.62	0.59–4.50	0.353
Positive nodes (0–3)	2.55	1.61–4.04	** < 0.001**	2.06	1.18–3.58	**0.011**
Histologic grade (1 vs. 2/3)	6.82	1.58–29.51	**0.010**	6.26	1.43–27.45	**0.015**
Age (≤ 50 vs. > 50 years)	0.83	0.33–2.07	0.687	–	–	–

	AMC (Korea)					
	Univariate			Multivariate		
	Hazard ratio	95% CI	*P* value	Hazard ratio	95% CI	*P* value
BCT risk (low vs. high)	7.26	2.94–17.90	** < 0.001**	4.22	1.46–12.19	**0.008**
Tumor size (≤ 2 cm vs. > 2 cm)	4.95	2.01–12.20	** < 0.001**	1.64	0.56–4.83	0.371
Positive nodes (0–3)	0.95	0.33–2.75	0.929	–	–	–
Histologic grade (1 vs. 2/3)	1.70	0.39–7.37	0.477	–	–	–
Age (≤ 50 vs. > 50 years)	4.58	1.52–13.81	**0.007**	2.60	0.79–8.56	0.115

AMC, Asan Medical Center; CI, confidence interval

*P* values < 0.05 are marked in bold

The prognostic value of the BCT score was also separately analyzed in the two cohorts. The median follow-up times of patients at Sagara Hospital and AMC were 11.9 and 18.6 years, respectively. Similar results were obtained in both cohorts. Significant differences in the 15-year DMFS between the BCT high-risk and low-risk groups were observed in both cohorts (Fig. 1b, c). In the Sagara Hospital cohort, the probability of 15-year DMFS was 90.2% (83.4–97.7%) for patients categorized as BCT low risk and 47.9% (28.3–80.8%) for those categorized as BCT high risk (Fig. 1b); the BCT high-risk group was significantly associated with a higher risk of 15-year distant metastasis (hazard ratio, 7.18; 95% CI, 2.88–17.89; *P* < 0.001) (Table 2). Similarly, in the AMC cohort, the patients categorized into the BCT high-risk group had a significantly shorter DMFS than those categorized into the BCT low-risk group (Fig. 1c), with the BCT high-risk group being a significant predictor of distant metastasis (hazard ratio, 7.26; 95% CI, 2.94–17.90; *P* < 0.001) (Table 2). Importantly, multivariate analysis revealed that the BCT high-risk group is an independent negative prognostic factor for 15-year DMFS in both the Sagara Hospital (hazard ratio, 3.92; 95% CI, 1.38–11.14; *P* = 0.010) and AMC (hazard ratio, 4.22; 95% CI, 1.46–12.19; *P* = 0.008) cohorts (Table 2).

### Prognostic ability of the BCT score to predict late recurrence 5–15 years after surgery

**Fig. 2:**
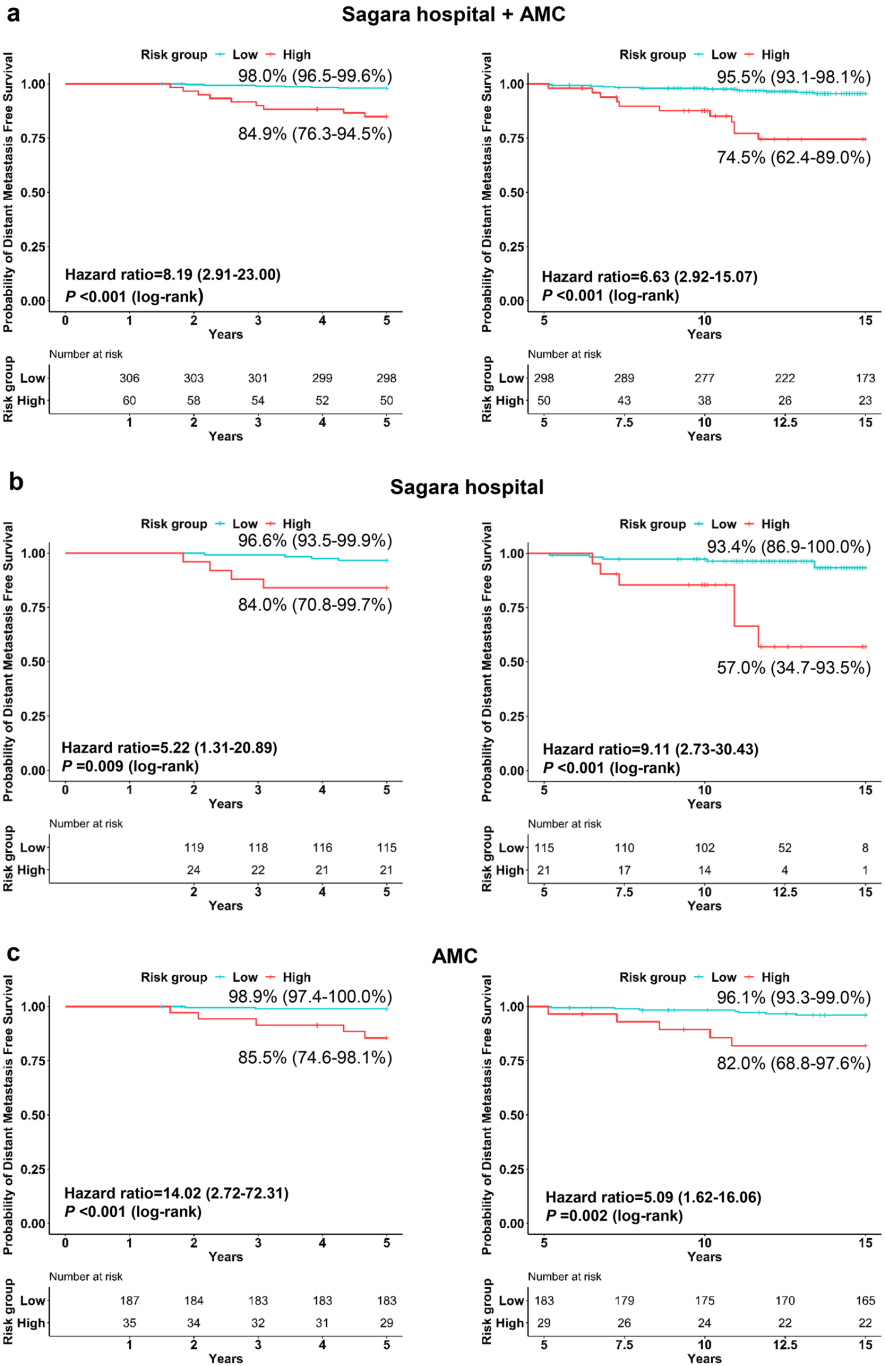
Kaplan–Meier plots of early and late recurrence in BCT high- and low-risk groups. Distant metastasis-free survival 0–5 (left) and 5–15 (right) years after surgery in patients in the **a** total, **b** Sagara Hospital (Japan), and **c** Asan Medical Center (AMC) (Korea) cohorts

We examined the ability of the BCT score to predict the risk of early (0–5 years) and late (5–15 years) recurrence. In the total cohort, Kaplan–Meier analyses showed that patients in the BCT high-risk group had a significantly lower probability of DMFS than those in the BCT low-risk group at both 0–5 years (*P* < 0.001) and 5–15 years (*P* < 0.001) (Fig. 2a). Among the 348 women who were distant metastasis-free at 5 years after surgery in the total cohort, the probability of 5–15-year DMFS was 95.5% (93.1–98.1%) for patients categorized into the BCT low-risk group and was 74.5% (62.4–89.0%) for those categorized into the BCT high-risk group. Multivariate analysis revealed that the BCT high-risk group is also an independent predictor of late recurrence (hazard ratio, 5.29; 95% CI, 1.99–14.09; *P* = 0.001) (Supplementary Table 1).

Similar results were observed in the Sagara Hospital and AMC cohorts when analyzed separately. In both cohorts, the 5-year and 5–15-year DMFS in the BCT high-risk group was significantly lower than that in the BCT low-risk group (Fig. 2b, c). Among patients who were distant metastasis-free within 5 years in both cohorts, the risk of 5–15-year distant metastasis was also significantly increased in patients categorized into the BCT high-risk group, and the BCT score was an independent predictor of 5–15-year DMFS (Supplementary Table 1). These results indicate that the BCT risk group stratification has prognostic value in predicting late distant recurrence in patients with HR-positive/HER2-negative early breast cancer.

We also compared the prognostic performance of the BCT score with traditional clinical prognostic factors to predict the risk of distant metastasis at 5–15 years. The clinical predictor was established by combining clinical prognostic factors for distant metastasis—tumor size, histologic grade, and nodal status—that were selected using the Cox regression analysis. In the total cohort, the BCT risk group significantly added prognostic information to the clinical prognostic factors for late recurrence (*P* = 0.003), whereas the clinical factors did not significantly add prognostic information to the BCT risk group (*P* = 0.758) (Table 3). Similar results were observed in both cohorts when analyzed separately (Table 3).

**Table 3:** Likelihood ratio analysis of the BCT score and clinical factors for 5–15-year distant metastasis-free survival

Site	Model 1	Model 2	LR Chi-square	*P* value
Sagara Hospital + AMC	BCT risk	BCT risk + clinical factors	1.18	0.758
	Clinical factors	Clinical factors + BCT risk	9.13	**0.003**
Sagara Hospital (Japan)	BCT risk	BCT risk + clinical factors	6.56	0.087
	Clinical factors	Clinical factors + BCT risk	4.13	**0.042**
AMC (Korea)	BCT risk	BCT risk + clinical factors	3.19	0.363
	Clinical factors	Clinical factors + BCT risk	4.40	**0.036**

AMC, Asan Medical Center; LR, likelihood ratio

Clinical factors: combination of tumor size, histologic grade, and positive nodes

### Prognostic value of the BCT score by age group

**Fig. 3:**
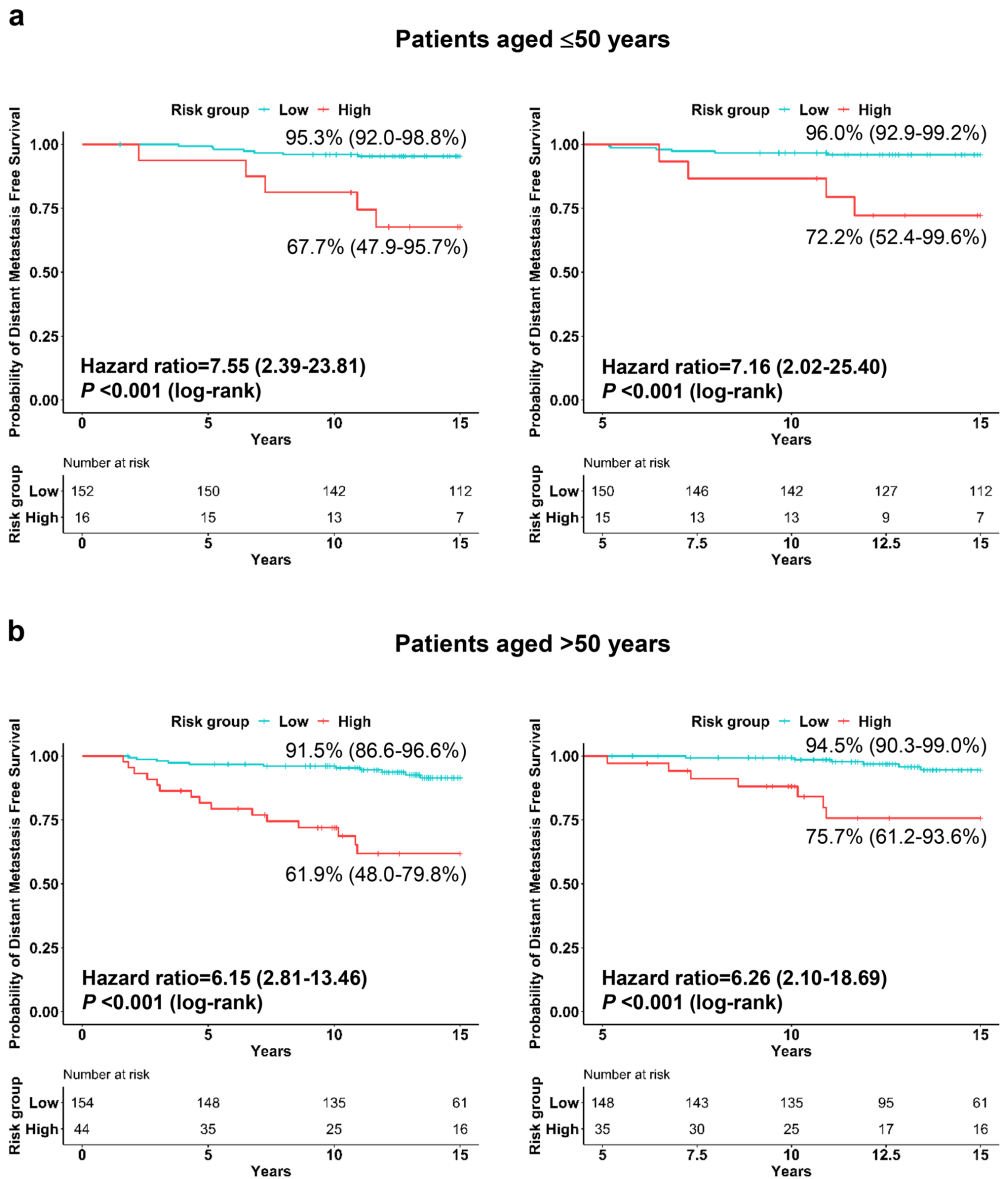
Kaplan–Meier plots of 15-year distant metastasis-free survival according to age group. Distant metastasis-free survival 0–15 (left) and 5–15 (right) years after surgery in **a** patients aged ≤ 50 years and **b** > 50 years in the total cohort

Finally, we assessed the prognostic value of the BCT score in patients aged ≤ 50 and > 50 years separately. The patient characteristics of each age group are described in Supplementary Table 2. Kaplan–Meier survival curves showed that the BCT high-risk group has a significantly shorter 15-year DMFS than the BCT low-risk group in both age groups (*P* < 0.001 and *P* < 0.001 in patients aged ≤ 50 and > 50 years, respectively) (Fig. 3). Multivariate analyses also revealed that the BCT high-risk group is independently associated with an increased risk of 15-year distant metastasis and 5–15-year distant metastasis in both age groups (Supplementary Table 3), indicating that the BCT score is a significant predictor of late recurrence regardless of age.

## Discussion

This study demonstrates that the GenesWell BCT assay can predict the risk of 15-year distant metastasis and identify patients at low risk for late recurrence from 5 to 15 years in Asian women with HR-positive/HER2-negative early breast cancer. Previous studies have reported the long-term prognostic significance of other multigene assays such as Oncotype DX [20], Prosigna [21], and EndoPredict [22]. However, their prognostic value has mainly been validated in patients from Western countries, being unclear whether they can predict the risk of 15-year recurrence in Asian populations. Our previous study comparing the BCT score and Oncotype DX 21-gene RS for risk classification showed a relatively low concordance between the two risk scores in Asian women aged < 50 years [23], supporting that multigene assays developed using data from patients with breast cancer mainly from Western countries may not be prognostic or predictive in Asian populations. This study is the first to validate the prognostic ability of the BCT score to predict 15-year distant metastasis in Asian women from Korea and Japan with HR-positive/HER2-negative early breast cancer.

Notably, this study validated the BCT score as a significant predictor of 15-year distant metastasis, including late recurrence 5–15 years after surgery, in Japanese women with early breast cancer. Although several multigene assays for breast cancer, such as Oncotype DX, MammaPrint, Prosigna, and EndoPredict, have been developed, only a few studies have evaluated their prognostic significance in the Japanese population. Toi et al. [24] showed that the Oncotype DX 21-gene RS has prognostic value in predicting 10-year distant recurrence in Japanese women with ER-positive/node-negative breast cancer. In addition, the 21-gene RS results have been reported to affect adjuvant treatment decision-making in Japanese women with ER-positive/HER2-negative early breast cancer [25]. However, the long-term prognostic significance of the 21-gene RS has not been evaluated in Japanese patients. Moreover, the 70-gene MammaPrint assay has been tested in Japanese women with early breast cancer, but its prognostic value is unclear [26, 27]. The multigene assay, Curebest 95GC Breast (Sysmex Corporation, Kobe, Japan), which measures the expression of 95 genes using DNA microarray, was developed in Japan [28] and is available for research purposes. A recent study validated that the 95GC high-risk group classified by this assay significantly predicts the risk of 10-year recurrence in ER-positive/node-negative breast cancer [29]. However, this was a retrospective study that included a small Japanese population, and the prognostic significance of the Curebest 95GC Breast was not evaluated for late recurrence beyond 10 years following surgery.

In the present study, subgroup analysis revealed that the BCT score has long-term prognostic value regardless of age group. We previously demonstrated that the BCT score is an independent significant predictor of 10-year recurrence in patients aged ≤ 50 years as well as in those aged > 50 years [19]. This study further revealed that the BCT high-risk group is a significant predictor of 15-year distant metastasis in both of these age groups. Given that the median and peak age of patients with breast cancer differ between Asian and Western countries [30] and also differ between Korean and Japan within Asia, as shown in this study, it is an important finding that the BCT score can predict the risk of late recurrence regardless of age. Hence, we suggest that the BCT score can be used to identify patients at low risk of recurrence who may not benefit from adjuvant chemotherapy, regardless of their age.

However, our study has several limitations, including a relatively small sample size and lack of subgroup analyses based on the nodal status of patients with breast cancer. As most patients with node-positive breast cancer received adjuvant chemotherapy, only a small number of node-positive patients were eligible for inclusion in this study; thus, we were unable to evaluate the prognostic value of the BCT score in predicting late recurrence at 5–15 years in these patients. A prospective study to evaluate the prognostic and predictive values of the BCT score is underway (ClinicalTrials.gov number NCT04278469).

## Conclusion

Our findings demonstrate the long-term prognostic value of the BCT score in predicting the risk of 15-year distant metastasis in Asian women with HR-positive/HER2-negative early breast cancer. This study also revealed that the BCT score predicts late recurrence after 5 years and is a valuable prognostic tool for both younger (≤ 50 years) and older (> 50 years) patients. These results indicate that the BCT score can identify patients at low risk of late recurrence who may not benefit from adjuvant chemotherapy or extend endocrine therapy, regardless of their age.

### Supplementary Information

Below is the link to the electronic supplementary material.

Supplementary file1 (DOCX 39 KB)

## Data Availability

All data relevant to the study are available from the corresponding author on reasonable request.
