# Supplementary material for: Long-term prognostic value of the GenesWell BCT score in Asian women with hormone receptor-positive/HER2-negative early breast cancer
Source: Breast Cancer. 2023 Oct 9;31(1):31–41. doi: 10.1007/s12282-023-01509-7 (PMC10764379; doi:10.1007/s12282-023-01509-7)
Supplement: Supplementary file 1 — Supplementary file1 (DOCX 39 KB) [file 12282_2023_1509_MOESM1_ESM.docx]

**Supplementary Tables**

**Supplementary Table 1.** Univariate and multivariate analyses for 5–15-year distant metastasis-free survival

|  | **Sagara Hospital + AMC** | | | | | | |
| --- | --- | --- | --- | --- | --- | --- | --- |
|  | **Univariate** | | |  | **Multivariate** | | |
|  | **Hazard ratio** | **95% CI** | ***P* value** |  | **Hazard ratio** | **95% CI** | ***P* value** |
| BCT risk (low vs. high) | 6.63 | 2.92–15.07 | **<0.001** |  | 5.29 | 1.99–14.09 | **0.001** |
| Tumor size (≤2 cm vs. >2 cm) | 2.64 | 1.17–5.99 | **0.020** |  | 1.31 | 0.52–3.34 | 0.568 |
| Positive nodes (0–3) | 1.89 | 1.10–3.22 | **0.020** |  | 1.26 | 0.66–2.43 | 0.484 |
| Histologic grade (1 vs. 2/3) | 1.74 | 0.59–5.12 | 0.315 |  | - | - | **-** |
| Age  (≤50 vs. >50 years) | 1.29 | 0.56–2.94 | 0.547 |  | - | - | - |
|  | **Sagara Hospital (Japan)** | | | | | | |
|  | **Univariate** | | |  | **Multivariate** | | |
|  | **Hazard ratio** | **95% CI** | ***P* value** |  | **Hazard ratio** | **95% CI** | ***P* value** |
| BCT risk (low vs. high) | 9.11 | 2.73–30.43 | **<0.001** |  | 6.61 | 1.83–23.83 | **0.004** |
| Tumor size (≤2 cm vs. >2 cm) | 2.90 | 0.88–9.52 | 0.080 |  | - | - | - |
| Positive nodes (0–3) | 3.15 | 1.69–5.87 | **<0.001** |  | 2.36 | 1.14–4.86 | **0.020** |
| Histologic grade (1 vs. 2/3) | 3.72 | 0.80–17.22 | 0.093 |  | - | - | **-** |
| Age  (≤50 vs. >50 years) | 0.52 | 0.16–1.71 | 0.280 |  | - | - | - |
|  | **AMC (Korea)** | | | | | | |
|  | **Univariate** | | |  | **Multivariate** | | |
|  | **Hazard ratio** | **95% CI** | ***P* value** |  | **Hazard ratio** | **95% CI** | ***P* value** |
| BCT risk (low vs. high) | 5.09 | 1.62–16.06 | **0.005** |  | 5.09 | 1.62–16.06 | **0.005** |
| Tumor size (≤2 cm vs. >2 cm) | 2.62 | 0.84–8.12 | 0.096 |  | - | - | - |
| Positive nodes (0–3) | 0.85 | 0.18–3.97 | 0.838 |  | - | - | - |
| Histologic grade  (1 vs. 2/3) | 1.02 | 0.22–4.66 | 0.979 |  | - | - | - |
| Age  (≤50 vs. >50 years) | 2.55 | 0.77–8.48 | 0.126 |  | - | - | - |
| Abbreviations : AMC, Asan Medical Center; CI, confidence interval | | | | | | | |
| *P* values < 0.05 are marked in bold. | |  |  |  |  |  |  |

**Supplementary Table 2.** Patient characteristics according to the age group

|  | **Sagara Hospital+AMC** | | | | |
| --- | --- | --- | --- | --- | --- |
|  | **Total** |  | **≤ 50 years** | **> 50 years** | ***P* value** |
| n (%) | 366 (100.0%) |  | 168 (100.0%) | 198 (100.0%) |  |
| Positive nodes |  |  |  |  | 0.073^b^ |
| 0 | 333 (91.0%) |  | 160 (95.2%) | 173 (87.4%) |  |
| 1 | 23 (6.3%) |  | 6 (3.6%) | 17 (8.6%) |  |
| 2 | 5 (1.4%) |  | 1 (0.6%) | 4 (2.0%) |  |
| 3 | 5 (1.4%) |  | 1 (0.6%) | 4 (20.0%) |  |
| Tumor size |  |  |  |  | **<0.001^a^** |
| ≤2cm | 300 (82.0%) |  | 155 (92.3%) | 145 (73.2%) |  |
| >2cm | 66 (18.0%) |  | 13 (7.7%) | 53 (26.8%) |  |
| Histologic grade |  |  |  |  | 0.531^a^ |
| 1 | 97 (26.5%) |  | 40 (23.8%) | 57 (28.8%) |  |
| 2 | 227 (62.0%) |  | 109 (64.9%) | 118 (59.6%) |  |
| 3 | 42 (11.5%) |  | 19 (11.3%) | 23 (11.6%) |  |
| BCT risk group |  |  |  |  | **0.002^a^** |
| Low | 306 (83.6%) |  | 152 (90.5%) | 154 (77.8%) |  |
| High | 60 (16.4%) |  | 16 (9.5%) | 44 (22.2%) |  |

Abbreviation: AMC, Asan Medical Center

^a^Chi-square test; ^b^Fisher’s exact test

*P* values < 0.05 are marked in bold.

**Supplementary Table 3.** Univariate and multivariate analyses for 15-year and 5–15-year distant metastasis-free survival according to age group

|  | **15-year distant metastasis-free survival** | | | | | | | | | | | | | | |  |
| --- | --- | --- | --- | --- | --- | --- | --- | --- | --- | --- | --- | --- | --- | --- | --- | --- |
|  | **Patients aged ≤50 years** | | |  |  |  |  |  | **Patients aged >50 years** | | |  |  |  |  | |
|  | **Univariate** | | |  | **Multivariate** | | |  | **Univariate** | | |  | **Multivariate** | | | |
|  | **Hazard ratio** | **95% CI** | ***P* value** |  | **Hazard ratio** | **95% CI** | ***P* value** |  | **Hazard ratio** | **95% CI** | ***P* value** |  | **Hazard ratio** | **95% CI** | ***P* value** | |
| BCT risk (low vs. high) | 7.55 | 2.39–23.81 | **0.001** |  | 6.09 | 1.53–24.26 | **0.010** |  | 6.15 | 2.81–13.46 | **<0.001** |  | 4.63 | 1.93–11.12 | **0.001** | |
| Tumor size (≤2 cm vs. >2 cm) | 4.64 | 1.25–17.19 | **0.022** |  | 1.75 | 0.36–8.53 | 0.491 |  | 3.61 | 1.67–7.81 | **0.001** |  | 1.90 | 0.80–4.51 | 0.147 | |
| Positive nodes (0–3) | 2.50 | 1.30–4.82 | **0.006** |  | 2.75 | 1.36–5.56 | **0.005** |  | 1.46 | 0.87–2.47 | 0.156 |  | - | - | - | |
| Histologic grade (1 vs. 2/3) | >1000 | 0.00–Inf | 0.998 |  | - | - | - |  | 2.27 | 0.78–6.59 | 0.132 |  | - | - | - | |
|  | **5** –**15-year distant metastasis-free survival** | | | | | | | | | | | | | | |  |
|  | **Patients aged ≤50 years** | | |  |  |  |  |  | **Patients aged >50 years** | | |  |  |  |  | |
|  | **Univariate** | | |  | **Multivariate** | | |  | **Univariate** | | |  | **Multivariate** | | | |
|  | **Hazard ratio** | **95% CI** | ***P* value** |  | **Hazard ratio** | **95% CI** | ***P* value** |  | **Hazard ratio** | **95% CI** | ***P* value** |  | **Hazard ratio** | **95% CI** | ***P* value** | |
| BCT risk (low vs. high) | 7.16 | 2.02–25.40 | **0.002** |  | 7.16 | 2.02–25.40 | **0.002** |  | 6.26 | 2.10–18.69 | **0.001** |  | 3.98 | 1.20–13.22 | **0.024** | |
| Tumor size (≤2 cm vs. >2 cm) | 1.02 | 0.22–4.80 | 0.981 |  | - | - | - |  | 4.80 | 1.48–15.59 | **0.009** |  | 2.87 | 0.79–10.47 | 0.109 | |
| Positive nodes (0 –3) | 2.00 | 0.79–5.07 | 0.142 |  | - | - | - |  | 1.91 | 0.96–3.80 | 0.067 |  | - | - | - | |
| Histologic grade (1 vs. 2/3) | >1000 | 0.00–Inf | 0.998 |  | - | - | **-** |  | 0.92 | 0.28–2.98 | 0.886 |  | - | - | **-** | |
| Abbreviation : CI, confidence interval | | | | | | | | | | | | | | | | |
| *P* values < 0.05 are marked in bold. | | | | | | | | | | | | | | | | |
